# Psychological factors and prosociality as determinants in grief reactions: Proposals for an integrative perspective in palliative care

**DOI:** 10.3389/fpsyg.2023.1136301

**Published:** 2023-03-28

**Authors:** Vittorio Lenzo, Maria C. Quattropani

**Affiliations:** Department of Educational Sciences, University of Catania, Catania, Italy

**Keywords:** clinical psychology, prolonged grief, prosociality, palliative care, health psychology

## 1. Introduction

The loss of a close family member is usually assumed to be one of the most stressful experiences. In their classical work, indeed, Holmes and Rahe (1967) classified the death of a spouse as the most stressful event, while the death of a close family member ranked fifth. Throughout the years, empirical research has examined the health outcomes of bereavement. One of the research's significant findings is that bereavement increases the risk of mortality, as well as the use of medical services (Stroebe et al., 2007). By way of understanding this finding, several risks and protective factors consisting of intrapersonal and interpersonal variables have been taken into account. For example, considering psychological resilience allow us to explain why some individuals develop a dysfunctional reaction, while other do not (Mancini and Bonanno, 2009). Although grief reactions are diverse, what is noteworthy here regards those leading to psychological symptoms. Depression and anxiety are commonly considered two of the most frequent symptoms among the bereaved, causing significant impairment in quality of life (Boelen and Prigerson, 2007). Among the diverse grief reactions, however, a pooled prevalence of 9.8% of the bereaved develop a syndrome named Prolonged Grief Disorder (PGD) (Lundorff et al., 2017), even though this finding was grounded on a variety of definitions. More recently, the prevalence rate 1 year after loss using the latest diagnostic DSM criteria for PGD was 4.4–10.9% (Prigerson et al., 2021). Another recent study found a prevalence rate of 3.3% when considering the DSM-5-TR criteria (Rosner et al., 2021). The Diagnostic and Statistical Manual of Mental Disorders, fifth edition, text revision (DSM-5-TR) included PGD in Section 2, under the chapter named “trauma- and stressor-related disorders” (American Psychiatric Association, 2022). The diagnostic criteria for PGD are the death of a loved one for at least 12 months and the onset of a chronic grief reaction characterized by deep yearning or longing for the lost person accompanied by preoccupation with thoughts or memories. Other symptoms characterizing PGD are identity disruption, intense emotional pain, and emotional numbness related to the loss. Since the high healthcare costs for individuals who develop a PGD, a preventive approach is paramount. This implies a deep understanding of protective and risk factors that might decrease or increase the likelihood of the onset of the disorder. Indeed, a glimpse into the factors underlying the risk of maladaptive reactions to grief is crucial. Of particular interest for empirical research and clinical practice is the field of palliative care. After the loss of their loved one, family members may experience intense grief reactions and, consequently, bereavement support represents a core component of palliative care. As argued by Lichtenthal (2018), improving screening and assessment of individuals at high risk of Prolonged Grief Disorder is challenging, along with implementing empirically supported interventions. However, it is hard to dispute that considering severe grief reactions as a mental disorder triggers some critical points. Research over the years has shown not only that a small but considerable percentage of individuals develop PGD after the death of a loved one, but that they may be at risk of stigmatization (Johnson et al., 2009; Gonschor et al., 2020). Thus, meeting the needs of bereaved individuals presents a substantial challenge to specialists within the field. Stigmatization toward individuals with PGD can have a detrimental impact on prevention as well as on seeking professional help (Schomerus and Angermeyer, 2008).

## 2. Bereavement and palliative care

In Italy, recent estimates reported more than 180.000 deaths for cancer (Italian Association of Medical Oncology, 2021), while almost 40.000 received palliative home care (Italian Ministry of Health, 2018). Family caregivers of patients in palliative care are more at risk of complicated grief (Guldin et al., 2011). World Health Organization. (2004) defines palliative care as “an approach that improves the quality of life of patients and their families facing the problem associated with life-threatening illness”. Relief from pain and other symptoms affecting a patient's quality of life represents the main aim of palliative care. Another aim concerns support for their family members after the loss, even though scant attention has been directed toward the prevention of complicated grief. By this token, it is not by chance that bereavement support has been designated as “the forgotten child” of palliative care (Hudson et al., 2018). Poor resources for individuals who need psychological support seem to characterize the public health approach to bereavement care (Aoun et al., 2012). Not all palliative services have a bereavement support program, even though prevention of complicated grief should represent the main target for them (World Health Organization., 2004). While on one hand, bereavement support remains under-researched, on the other hand, professionals still refer to past theories lacking empirical evidence (Bonanno, 2009). At best, some of these past theories have conceived grief as a sequence of phases that must be going through identically to yield the most efficacy results. Nevertheless, the needs of informal caregivers of patients in palliative care are more complex than expected by a model of the stages of grief, especially when they struggle with the loss. In this vein, it could be useful to consider trajectories of grief over time. For instance, in a study involving a sample of 28 recent widows, Bisconti et al. (2006) found that the pattern of emotional wellbeing widely oscillated across 3 months. Put another way, confusion about one's emotional state may characterize the period immediately following the loss. Factors such as social support may influence the overall intraindividual trend. Another caveat regards antecedent predictors of trajectories of grief. Bonanno et al. (2002) conducted a prospective study from preloss to 18 months postloss among a sample of 205 individuals belonging to the general population. Unlike what one might expect, the resilient pattern outlined by low depression levels over time was the most frequent. Instead, relative to other patterns, common grief pattern was infrequent enough. These findings are in line with Bonanno's (2004) review, indicating that most people facing a potentially traumatic experience showed resilience. Despite there is still lack of evidence in the context of palliative care, some research has attempted to get a glimpse into the psychological factors related to the risk of grief disorder. As one way to better grasp the considerable variation in grief reactions, several authors have zeroed in on the crucial role of attachment style (Bowlby, 1969). Indeed, the attachment system may be undermined by the loss of a loved one. For example, in a study including a sample of 157 family caregivers of cancer patients assisted in palliative home care, Lenzo et al. (2022a) found that discomfort with closeness and relationship as secondary, which are two dimensions of the high order factor of avoidance attachment, moderated the effect of the perceived support on the severity of grief symptoms. Another relevant finding of this study was the need to consider together psychological and social factors. So doing this might then offer a way to grasp the interplay among variables responsible for grief reaction, which can improve the efficacy of any psychological intervention.

## 3. Discussion

**Figure 1:**
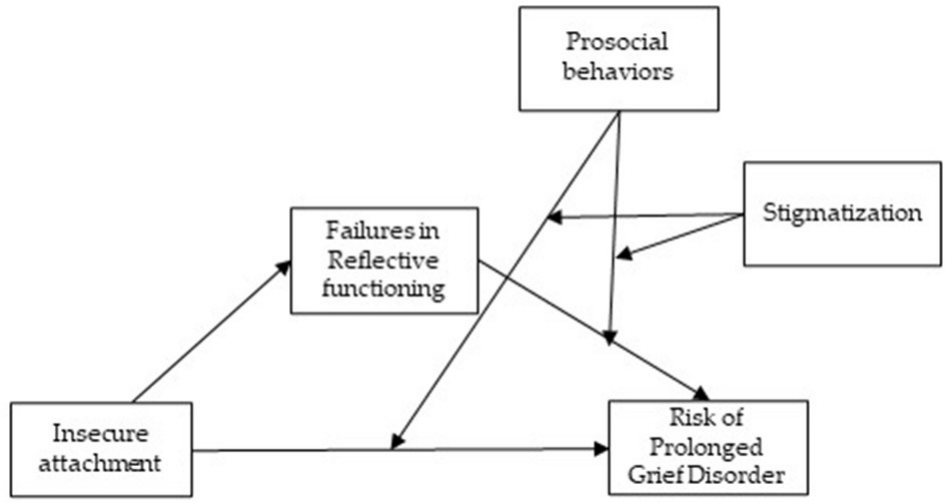
Conceptual model.

A lot of attention has been directed toward understanding psychological factors underneath grief reactions. However, bereavement theories identifying hypothetical stages of mourning have left unanswered many important questions. A major limitation of this kind of theory is the idea that grief resolution progressed in a linear fashion (Rothaupt and Becker, 2007). The evidence seems to offer a different outline of the factors explaining grief reactions. First of all, contrary to what can be expected, most of the people facing potentially traumatic events showed psychological resilience (Mancini and Bonanno, 2009). Moreover, grief reactions tend to be more complex than what the linear model can explain. Another major limitation concerns the risk of pathologizing grief by introducing a mental disorder related to grief (American Psychiatric Association, 2022). Since its introduction, a considerable debate has characterized literature, though most people agreed that Prolonged Grief Disorder could be deemed a mental disorder (Tang et al., 2020). Friends or relatives of family caregivers may stigmatize symptoms experienced by the bereaved (Johnson et al., 2009), though engagement in activities to help them is frequent. Any intentional behavior aimed to benefit another is defined as “prosocial” (Dunfield, 2014; Geraci and Franchin, 2021). Prosocial behaviors appear early in development and play a fundamental role during the life span of humans. There is evidence that prosocial behavior fosters social support, promotes resilience, and improves mental health as well as the quality of life (Ramkissoon, 2022). Yet, prosocial behavior may arise from positive psychological conditions as well as negative ones (Vollhardt, 2009). Although research has mostly focused on its development, prosocial behaviors may play a role in grief reactions among the bereaved. Prevention of PGD, indeed, may be fostered by prosocial behavior around the bereaved caregivers. For example, family associations composed of individuals who had similar experiences could help the bereaved caregivers by sharing coping strategies. Also, other prosocial behaviors offered by family associations such as comforting those who are hurt can alleviate emotional distress and reduce the fear of stigmatization. In this research field, understanding the relationships between these variables remains challenging and more studies are needed. Research has already demonstrated that personality traits are associated with regulations of social behavior (Fiddick et al., 2016). Yet, the possible presence of psychopathology can decrease moral judgment as well as engage in prosocial behavior (Memmott-Elison and Toseeb, 2022). Besides increasing our comprehension of grief reactions, knowledge of prosociality would have much other practical value. Several studies converged in suggesting that prosocial behaviors might have a positive impact on stress (Brown and Brown, 2015; Raposa et al., 2016; Lazar and Eisenberger, 2022). Undoubtedly, prosocial behaviors in response to negative states such as helping, sharing, caring, and comforting may alleviate emotional distress. As already stated, high levels of insecure attachment seem to be associated with the severity of symptoms (Lenzo et al., 2022a). Likewise, low levels of certainty about mental states tend to be related to more symptoms of anxiety and depression (Lenzo et al., 2022b). With few exceptions (Carr et al., 2001; Stroebe et al., 2005), when considering family caregivers of patients deceased in palliative care, social support is paramount (Logan et al., 2018). The relationships between personality traits and prosocial behaviors might be meaningful but not straightforward. In pursuing this question, it could be reasonable to hypothesize that prosocial behavior plays as a moderator of the relationship between insecure attachment and failures in mentalizing, on one hand, and the risk of PGD, on the other hand (Figure 1). At the very least, a deeper understanding of the pathways from individual differences in personality traits to prosocial behavior may be able to lead to more efficacy psychological interventions (Caprara et al., 2012), especially at a time like this. The COVID-19 pandemic has brought to the fore the relevance of prosocial behavior. Findings of several studies have hitherto well-established the impact of the pandemic on mental health among people (Lenzo et al., 2022c,d), even though protective factors such as resilience contribute to lower it (Lenzo et al., 2020). The COVID-19 pandemic also offers an unprecedented opportunity to grasp the mechanisms underneath prosociality and loss. For instance, restrictive measures and social distancing following the spread of contagion have undoubtedly increased loneliness, which increases negative consequences for mental and physical health (Ernst et al., 2022). Still, restrictive measures in hospitals or funerals without the presence and held remotely may increase the risk of PGD (Wallace et al., 2020). In this vein, prosocial behavior is of utmost relevance to mitigate the potential negative effect of the pandemic. In conclusion, more attention to the underlying psychological factors of negative grief reactions, as well as to the interplay between social behaviors and bereavement, would represent an encouraging approach to prevent PGD among family caregivers of patients deceased in palliative care.

## Author contributions

All authors listed have made a substantial, direct, and intellectual contribution to the work and approved it for publication.
